# Reliability of Interleukin-6 Alone and in Combination for Diagnosis of Early Onset Neonatal Sepsis: Systematic Review

**DOI:** 10.3389/fped.2022.840778

**Published:** 2022-03-23

**Authors:** Julia Eichberger, Bernhard Resch

**Affiliations:** ^1^Research Unit for Neonatal Infectious Diseases and Epidemiology, Medical University of Graz, Graz, Austria; ^2^Division of Neonatology, Department of Pediatrics and Adolescent Medicine, Medical University of Graz, Graz, Austria

**Keywords:** interleukin-6 (IL-6), early onset neonatal sepsis, diagnostic accuracy, sensitivity and specificity, meta-analysis

## Abstract

Neonatal sepsis is a major cause of morbidity and mortality in both preterm and term infants. Early-onset neonatal sepsis (EONS) presents within the first 72 h of life. Diagnosis is difficult as signs and symptoms are non-specific, and inflammatory markers are widely used to confirm or rule out neonatal sepsis. Interleukin-6 (IL-6) is part of the fetal inflammatory response syndrome (FIRS) and therefore an interesting early marker for neonatal sepsis. The main objective for this review was to assess the diagnostic potential of IL-6, alone and in combination, for diagnosis of early neonatal sepsis (EONS) in term and preterm infants, in cord and peripheral blood, and in dependence of timing of sample collection. IL-6 diagnostic accuracy studies for diagnosing EONS published between 1990 and 2020 were retrieved using the PubMed database. We included 31 out of 204 articles evaluating the potential of IL-6 for the diagnosis of EONS in a study population of newborns with culture-proven and/or clinically suspected sepsis. We excluded articles dealing with neonatal bacterial infections other than sepsis and biomarkers other than inflammatory markers, those written in languages other than English or German, studies that did not distinguish between EONS and late-onset sepsis, and animal and *in vitro* studies. Full-text articles were checked for other relevant studies according to the PRISMA criteria. We identified 31 studies on IL-6 diagnostic accuracy for EONS diagnosis between 1990 and 2020 including a total of 3,276 infants. Sensitivity and specificity were reported, and subgroup analysis was performed. A STARD checklist adapted for neonates with neonatal sepsis was used for quality assessment. The range of IL-6 sensitivity and specificity in neonatal samples was 42.1–100% and 43–100%; the median values were 83 and 83.3%, respectively. IL-6 accuracy was better in preterm infants than in mixed-study populations. Early sample collection at the time of sepsis suspicion had the highest sensitivity when compared to other time points. Cord blood IL-6 had higher diagnostic value compared to peripheral blood. The biomarker combination of IL-6 and CRP was found to be highly sensitive, but poorly specific. Limitations of this review include use of only one database and inclusion of a heterogeneous group of studies and a small number of studies looking at biomarker combinations; a strength of this review is its focus on early-onset sepsis, since type of sepsis was identified as a significant source of heterogeneity in IL-6 diagnostic accuracy studies. We concluded that IL-6 has a good performance as an early diagnostic marker of EONS within a study population of preterm infants, with best results for cord blood IL-6 using cutoff values above 30 pg/ml.

## Introduction

Neonatal sepsis is still one of the leading causes of morbidity and mortality in the neonatal intensive care unit (NICU) (1). The symptoms are variable and non-specific (2). Diagnosis and treatment of neonatal sepsis remain challenging (3). Early and efficient treatment is crucial for a good neonatal outcome and prognosis in neonatal sepsis cases often necessitating empirically selected broad-spectrum antibiotics in high-risk infants (4, 5). Empirical treatment, however, increases the exposure to adverse drug effects and nosocomial complications and contributes to the development of resistant strains (6). For the United States, it has been shown that for every neonate with proven bacterial sepsis, 11–30 infants with negative sepsis status receive antibiotics (7). Withholding or delaying treatment in a potentially infected child, however, would be inacceptable given the rapid course and high fatality associated with neonatal sepsis (8). Biological markers that react rapidly after the onset of the inflammatory process are greatly needed in the diagnosis of neonatal sepsis (9).

Interleukin-6 (IL-6) is characterized by a short half-life due to binding to plasma proteins such as α2-macroglobulin, early storage in the liver, or inhibition by other cytokines (10). The cytokine IL-6 is a particularly early marker of neonatal sepsis. It is released within 2 h after the onset of bacteremia, peaks at approximately 6 h, and finally declines over the following 24 h (11). IL-6 levels are significantly elevated up to 48 h prior to the onset of clinical sepsis (12). While some investigators have found that the neonatal IL-6 response is comparable to that found in adults, others have reported a diminished IL-6 production (2, 13). Stress and tissue injury have the potential to provoke an IL-6 response (14, 15). Interpretation of IL-6 levels for diagnosis of neonatal sepsis might therefore be hampered by underlying illnesses and their severity. To improve the diagnostic capacity of this early marker, combinations with later and more specific biomarkers (e.g., CRP) have been suggested (16). A relatively large sample size is required since IL-6 circulates at rather low levels (17).

Chiesa et al. (18) studied the upper reference limits and dynamics of IL-6 over the first 48 h of life in 148 healthy babies (113 term, 35 near-term). Samples were obtained at three fixed neonatal ages (0, 24, and 48 h after birth). The geometric mean IL-6 concentrations in the healthy term babies were 1.69 at birth, 4.09 at 24 h, and 3.45 pg/ml at 48 h of life. Healthy near-term babies had corresponding IL-6 values of 10.9, 9.3, and 8.4 pg/ml (18).

IL-6 is one of the most studied cytokines in sepsis; its circulating levels rise rapidly in response to infection and are closely associated with sepsis prognosis and mortality in adults (2, 5). For septic neonates, divergent results have been published, ranging from diminished IL-6 production in term and even more pronounced in preterm infants to IL-6 concentrations comparable to that found in adults (2). In neonates, IL-6 is an early and highly sensitive marker (4, 19). Interestingly, IL-6 levels in cord blood correlate well with neonatal hematologic indices used to evaluate EONS (8). However, the specificity of IL-6 is often low (4), and increased IL-6 values were also found in infants with non-infectious conditions limiting its use in the differentiation of neonates having infections or not (14).

A crucial factor for the implementation of inflammatory markers for neonatal sepsis diagnosis seems to be the difficulty to formulate a definitive opinion on their clinical usefulness from the findings of current literature (15). Small sample sizes, inconsistent definitions of sepsis, heterogeneity of the study population, and differences between cutoff values led to inconclusive results in diagnostic accuracy studies (20). Mehr et al. (21) stated, back in 2000, that the heterogeneous methods of laboratory measurement and the wide variations in data analysis and in reporting results precluded the possibility of performing a meaningful meta-analysis—problems that remain an issue even today (20). Either reliable cutoff values are lacking or there is an abundance of different cutoffs proposed for the same marker, both rendering a potential diagnostic test wearisome to apply clinically (15). The aim of the study was to determine the actual role of IL-6 alone or in combination for the diagnosis of EONS by means of a meta-analysis including studies from 1990 to 2020, to identify factors that possibly affect the diagnostic potential of IL-6 and investigate them by means of a subgroup analysis.

## Methods

Studies eligible for review inclusion were retrieved using the PubMed database including diagnostic accuracy studies of IL-6 in neonates published between 1990 and 2020. The combined search term used was (Interleukin-6 OR IL-6) AND (neonatal sepsis OR neonatal infection OR sepsis) AND (early-onset sepsis OR EOS OR EONS). No PubMed filters or language restrictions were used.

**FIGURE 1:**
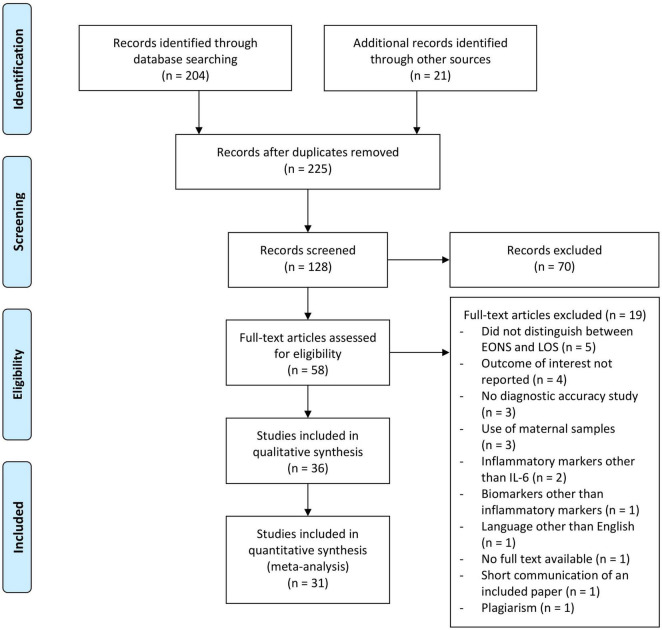
Flowchart of the study selection process for diagnostic accuracy of interleukin-6 in early onset neonatal sepsis between 1990 and 2020.

Reviewer (JE) conducted the database search and identified potential studies by screening titles and abstracts. For inclusion, the following criteria had to be fulfilled on an abstract level: the study population consisted of newborns, the subjects presented with culture-proven and/or clinically suspected sepsis, and the article evaluated the potential of IL-6 (alone or in combination with other inflammatory markers) for the diagnosis of early-onset neonatal sepsis. Excluded were articles dealing with other neonatal bacterial infections, those written in languages other than English or German, studies that did not distinguish between EONS and late-onset sepsis, animal and *in vitro* studies, and studies analyzing biomarkers other than inflammatory markers. Subsequently, full-text articles of shortlisted studies were assessed for eligibility (JE). Reference lists of obtained articles and relevant review articles were hand searched for other relevant studies according to the PRISMA criteria (see Figure 1). In cases of doubt, study eligibility was resolved with input from an independent reviewer (BR). Relevant data from the eligible studies were extracted by reviewer JE using a standardized data collection form. Data extraction included the following: first author, country, year of publication, sepsis definition, and number and specific characteristics of the newborns in the septic and non-septic groups (summarized under recruitment). Further data included reference standards employed, sample studied, time of sample collection, whether IL-6 was used alone or in combination with another inflammatory marker, and finally the test method used. The analyses were based on previously published studies. Therefore, no patient consent, ethical approval, and institutional review board were required.

Reviewer JE assessed the quality of reporting of the included studies using a data extraction form based on the STARD checklist and adapted for neonates with neonatal sepsis by Chiesa et al. (22). The key domains—descriptions of participant recruitment, reference standard and index test, and study population—are evaluated simply answering yes, if the item is present, or no if not (22).

Statistics included evaluation of studies regarding sensitivity and specificity of IL-6, the cutoff value, and the area under the curve (AUC) of IL-6 for sepsis diagnosis. The sensitivity indicates the percentage of patients with sepsis diagnosis who had a plasma cytokine level above the given cutoff, specifically the percentage of patients not meeting the criteria of sepsis who had cytokine levels below the cutoff value. Box-plot diagrams were used to show the distribution of cutoff levels used and sensitivity and specificity values reported, while forest plots show sensitivity and specificity values of the individual studies as well as pooled sensitivity and specificity. The 95% confidence intervals were calculated using Wilson’s method (23).

By performing extensive literature research, we identified factors that have been shown to influence IL-6 levels in neonates, namely, gestational age, type of sample, time of sample collection, and choice of cutoff value. Since those factors represent possible causes of heterogeneity in diagnostic accuracy studies of IL-6, we subsequently researched their influence on study results by subgroup analysis within our meta-analysis. Pooled sensitivities and specificities were calculated by grouping studies, coinciding with influencing factors together, and treating their infants as one big study population. To investigate the influence of gestational age, subgroups were formed by grouping all preterm infants and all infants from mixed (preterm and term) study populations together. To further evaluate the influence of a low vs. a high cutoff value, these subgroups were again divided by the median cutoff level present within the respective subgroup. The influence of type of sample was investigated by forming subgroups of cord versus peripheral blood samples. The peripheral blood sample subgroup was further divided into those studies reporting sample collection times earlier than 48 h of life (e.g., ≤ 6 h, ≤ 12 h) and studies allowing larger time intervals for sample collection (up to 1 week). Meta-analysis on a certain biomarker combinations was performed if at least 3 studies studied this combination.

## Results

As shown in Figure 1, 204 records on IL-6 as a marker of EONS were identified from the PubMed database search and 21 additional records were identified by screening the reference lists of included articles and relevant review articles (22, 24–27). Following exclusion of studies by titles, 128 abstracts were screened. The full text of 58 articles was assessed, and finally 31 studies including 3,276 infants were eligible for meta-analysis.

Most studies agreed on the definition of EONS, as sepsis occurring before 72 h of life (4–6, 8, 19, 20, 28–32). Other definitions included sepsis ≤ 48 h (*n* = 5) (33–37), sepsis ≤ 1 week (*n* = 1) (38), sepsis ≤ 5 days (*n* = 1) (9), sepsis ≤ 4 days of life (*n* = 1) (39), and sepsis within the first days of life (*n* = 1) (40). Eleven studies did not specify their definition (2, 3, 7, 10, 14, 17, 41–45).

**FIGURE 2:**
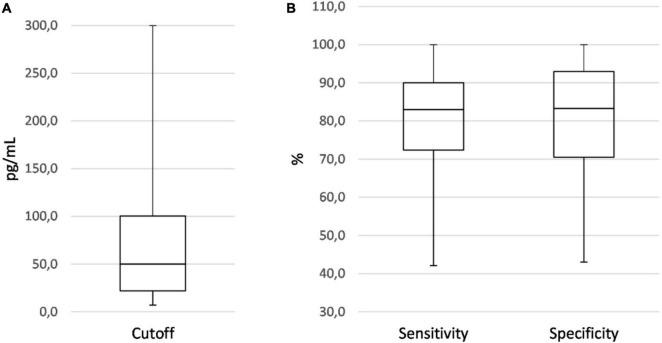
Boxplots showing the distribution of IL-6 cutoff **(A)**, sensitivity, and specificity values **(B)** of all diagnostic accuracy studies on EONS using neonatal samples.

**FIGURE 3:**
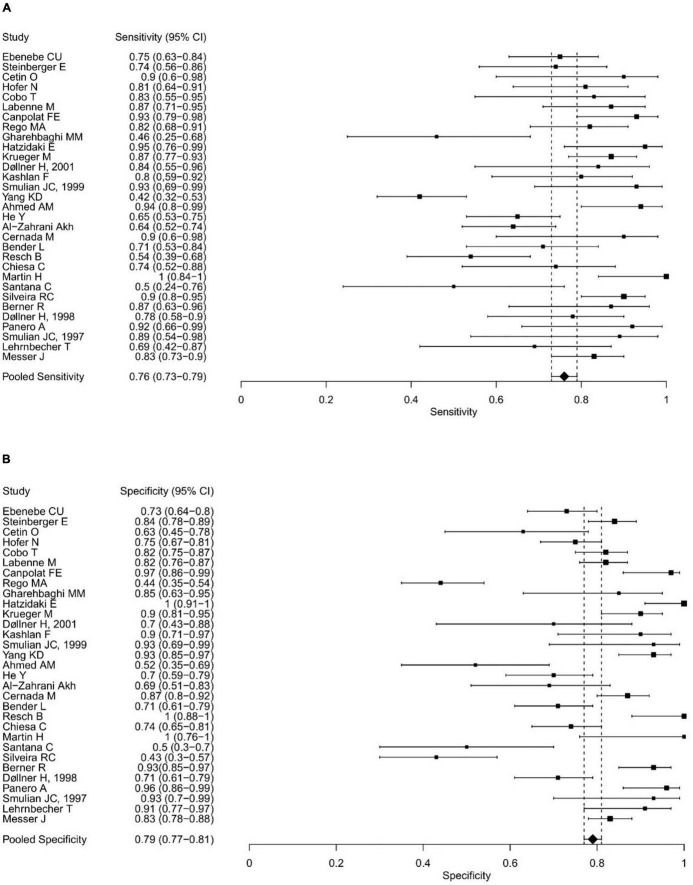
Forest plots showing the individual and pooled sensitivities **(A)** and specificities **(B)** of IL-6 diagnostic accuracy studies for the diagnosis of EONS.

The range of IL-6 sensitivities and specificities was 42.1–100% and 43–100%, respectively; the median values were 83 and 83.3% (see boxplots in Figure 2). Pooled sensitivity was 76% (95% CI: 73–79%), pooled specificity 79% (77–81%) (see forest plots in Figure 3). The data extracted from the included IL-6 diagnostic accuracy studies is summarized in Table 1 for preterm infants, in Table 2 for mixed study populations, and in Table 3 for IL-6 in combination with other inflammatory markers.

**TABLE 1:** Characteristics of IL-6 accuracy studies for diagnosing EONS in the preterm infant.

Author, year, country, (reference)	EONS definition	Recruitment	Reference standard in infected neonates	Reference standard in control neonates	Sample studied, time of sample collection	Test	IL-6 cutoff (pg/mL)	Sensitivity, % (95% CI)	Specificity, % (95% CI)	AUC (95% CI)	PPV (%)	NPV (%)
Ebenebe et al., Germany, (4)	≤72 h	182 preterm infants with a birth weight <2,000 g: 67 infected, 115 uninfected	(1) Positive blood culture or (2) CRP ≥ 5 mg/l and ≥ 3 clinical signs	Gestational age and birth-weight matched neonates that did not fulfill criteria of EONS	Neonatal blood, 0 h (PNA)	Electrochemi luminescence assay	40	75	72.8	0.804	14	98
Steinberger et al., Austria, (17)	NS	218 NICU preterm infants with risk factors for EONS: 30 infected, 188 uninfected	(1) Positive blood culture or (2) ≥3 categories of clinical signs or (3) ≥1 categories of clinical signs, and ≥2 laboratory abnormalities (CRP, WBC, I:T ratio)	NA	Cord blood, 0 h (PNA)	ELISA	15.85 (ROC, Youden)	73.7 (51.2–88.2)	84.2 (75.8–90)	0.812 (0.675–0.948)	46.7 (30.2–63.9)	94.4 (87.6–97.6)
Cetin et al., Turkey, (41)	NS	40 preterm infants born to mothers with pPROM: 10 infected, 30 uninfected	Positive blood or gastric washing culture and/or clinical findings	NA	Cord blood, 0 h (PNA)	ELISA	11 (ROC, NS)	90 (55–98)	63.3 (43–80)	0.767 (0.608–0.926)	45	95
Hofer et al., Austria, (28)	≤72 h	176 preterm infants at risk of bacterial infection: 32 EONS, 144 other	(1) Positive bacterial culture from umbilical cord blood, peripheral blood, or CSF or (2) negative culture, but ≥3 categories of clinical sepsis signs, with either ≥1 maternal risk factors or ≥2 abnormal laboratory markers (CRP, WBC, I:T ratio)	NA	Cord blood (UV), 0 h (PNA)	ELISA	11.1 (ROC, Youden)	81	75	0.795 (0.695–0.896)	NA	NA
Cobo et al., Czech Republic, (6)	≤72 h	176 preterm infants born to mothers with PPROM: 12 infected, 164 uninfected	(1) Positive blood culture or (2) clinical signs and ≥2 abnormal hematological laboratory results (WBC, PC, I:T ratio)	NA	Cord blood, 0 h (PNA)	ELISA	38 (ROC, NS)	83	82	0.908 (0.846–0.971)	30	98.1
Labenne et al., France, (7)	NS	213 NICU preterm infants with a presumptive diagnosis of EONS: 31 infected, 182 uninfected	(1) Positive culture of blood or CSF, and clinical signs or (2) clinical signs, CRP >1 mg/dl, positive superficial or placental cultures, and no alternative diagnosis	(1) Positive superficial culture without abnormal CRP or (2) CRP > 1 mg/dl and an alternative diagnosis or (3) neither positive culture nor abnormal CRP	Venous blood, at sepsis evaluation (≤6 h PNA)	Cytometric bead array (a multiplexed system)	300 (ROC, minimizing number of misclassified episodes)	87.1 (71.1–94.9)	82	0.895 (0.837–0.953)	NA	97.3
Canpolat et al., Turkey, (40)	Within the first days of life	74 preterm infants born to mothers with pPROM: 32 infected, 42 uninfected	(1) Positive blood culture and clinical signs and/or abnormal laboratory findings or (2) negative blood culture, but clinical and/or laboratory findings	Negative blood culture and no clinical or laboratory findings	Cord blood (UV), 0 h (PNA)	ELISA	7.6 (ROC, NS)	93	96.7	NA	NA	NA
Rego et al., Brazil, (42)	NS	144 NICU preterm infants presenting RDS during the first 24 h of life: 44 infected, 100 infected	In addition to RDS, (1) ≥2 categories of clinical signs, or clinical chorioamnionitis, and positive blood or CSF culture or (2) ≥ 2 categories of clinical sepsis, or clinical chorioamnionitis, and a hematologic sepsis score > 3 or 3) radiographic evidence of pneumonia and a hematologic sepsis score > 3	No clinical signs and a hematologic sepsis score <3	Peripheral blood, 0 h (from suspicion of sepsis)	Chemilu minescence immunoassay	36 (ROC, maximum sensitivity and specificity >50%)	82 (67–93)	44 (33–55)	0.72 (0.62–0.83)	40 (29–51)	85 (71–94)
Gharehbaghi et al., Iran, (29)	≤72 h	45 NICU preterm infants born to mothers with PROM: 17 infected, 18 uninfected	(1) Positive blood culture or (2) negative blood culture, but ≥3 clinical signs of sepsis associated with laboratory findings (WBC, platelet count, I:T ratio)	NA	Cord blood, 0 h (PNA)	ELISA	20	46	85	NA	88	39
Hatzidaki et al., Greece, (43)	NS	58 preterm neonates born to mothers with pPROM: 20 infected, 38 uninfected	(1) Positive blood culture within 4 days of life or (2) ≥3 categories of clinical signs and ≥2 abnormal laboratory findings	NA	Cord blood, 0 h (PNA)	ELISA	108.5 (ROC, NS)	95	100	NA	100	97.4
					Neonatal blood, on day 4 (PNA)	ELISA	55 (ROC, NS)	90	97.4	NA	94.7	94.9
Krueger et al., Germany, (34)	≤48 h	Of the 136 infants, 77 were preterm: 40 infected, 37 uninfected	(1) Clinical signs and positive blood culture or (2) clinical signs and abnormal laboratory results (CRP, I:T ratio), biological fluids positive for bacteria, or signs of inflammation in placenta	Non-infectious clinical conditions	Cord blood, 0 h (PNA)	Fully automated chemil uminescence immunoassay	80 (ROC, ULC)	96	94	NA	NA	NA
Døllner et al., Norway, (44)	NS	24 NICU preterm infants: 11 infected, 13 uninfected	(1) Clinical signs, and a positive blood culture or (2) ≥3 categories of clinical signs, and CRP ≥ 3 mg/dl or (3) radiographic evidence of pneumonia, respiratory signs or symptoms, and CRP ≥ 3 mg/dl	Clinical conditions apparently non-infectious	Cord blood, 0 h (PNA)	ELISA (Quantikine)	33	84	70	0.86 (0.66–0.96)	NA + L9:O12	NA
Kashlan et al., United States, (3)	NS	43 NICU singleton, very preterm infants (≤32 weeks GA): 21 infected, 22 uninfected	(1) Positive culture of blood and/or CSF or (2) ≥3 maternal/neonatal indicators for infection (risk factors, clinical signs, abnormal hematological findings)	Negative blood culture and <3 maternal/neonatal indicators for infection	Cord blood (UV), 0 h (PNA)	Enzyme-linked immunoassay (Endogen)	100 (ROC, NS)	80	90	NA	89	83
Smulian et al., United States, (30)	≤72 h	28 preterm infants with either spontaneous preterm labor or PPROM: 14 infected, 14 uninfected	(1) Autopsy or positive CSF or blood culture or (2) clinical signs and ≥2 laboratory abnormalities (WBC, I:T ratio, PC, abnormal CSF analysis)	NA	Cord blood (UV), 0 h (PNA)	ELISA (Quantikine)	25 (ROC, ULC)	92.9	92.9	NA	92.9	92.9

*NS, bot specified; NA, bot available to NS, not specified; NA, not available; UV, umbilical vein; UA, umbilical artery; PNA, postnatal age; NICU, neonatal intensive care unit; CSF, cerebrospinal fluid; CRP, C-reactive protein; WBC, white blood count; PC, platelet count; ABC, absolute band count; EONS, early-onset neonatal sepsis; AUC, area under the curve; PPV, positive predictive value; NPV, negative predictive value; GA, gestational age.*

**TABLE 2:** Characteristics of IL-6 accuracy studies for diagnosing EONS in a mixed population of preterm and term infants.

Author, year, country, (reference)	EONS definition	Recruitment	Reference standard in infected neonates	Reference standard in control neonates	Sample studied, time of sample collection	Test	IL-6 cutoff (pg/mL)	Sensitivity, % (95% CI)	Specificity, % (95% CI)	AUC (95% CI)	PPV, %	NPV, %
Yang et al., China, (5)	≤72 h	152 preterm (>34 weeks) and term infants at risk for EONS: 76 infected, 76 uninfected	(1) Positive blood or CSF culture or (2) ≥3 categories of clinical signs	Negative blood culture and <3 categories of clinical signs	Venous blood, ≤72 h (PNA)	MILLIPLEX Map Human Th17 Magnetic Bead Panel and Sepsis Panel (Millipore)	153	42.1	93.4	0.704 (0.622–0.786)	84.6	61.4
Ahmed et al., Egypt, (31)	≤72 h	60 NICU preterm and term infants: 30 high suspicion of EONS, 30 matched controls	Clinical findings supporting the suspicion of neonatal sepsis	Age- and weight-matched neonates without the criteria of sepsis suspicion	Venous blood, ≤72 h (PNA)	ELISA	24 (ROC, Youden)	94.4	52.4	0.751 (0.623–0.854)	45.9	95.7
He et al., China, (19)	≤72 h	151 preterm (>34 weeks) and term infants with suspected EONS: 68 infected, 83 uninfected	(1) Positive blood or CSF culture and any abnormal finding or (2) negative culture results but ≥3 abnormal findings	Negative culture results and <3 abnormal findings	Venous blood, ≤72 h (PNA)	MILLIPLEX Map Human Th17 Magnetic Bead Panel and Sepsis Panel (Millipore)	75.43	64.71	69.88	0.706 (0.626–0.777)	63.77	70.74
Al-Zahrani et al., Saudi Arabia, (38)	<1 week	100 NICU preterm and term infants with suspected sepsis: 71 infected, 29 uninfected	(1) Positive blood culture and/or positive PCR results for bacterial 16S rDNA or (2) negative blood culture and PCR, but clinical signs of sepsis and positive sepsis screen.	Neonates suspected of having sepsis with negative blood culture, PCR and sepsis screen	Blood sample, ≤24 h (after NICU admission), <1 week (PNA)	ELISA	60	63.6	69	NA	75.6	55.5
Cernada et al., Spain, (20)	≤72 h	128 preterm and term infants with prenatal risk factors for EONS (77% asymptomatic at birth): 10 infected, 118 uninfected	(1) Positive blood culture and clinical sings or (2) ≥3 categories of clinical signs	NA	Cord blood, 0 h (PNA)	Chemiluminescence enzyme immunoassay in solid phase	255.87 (ROC, NS)	90	87.4	0.88 (0.7–1.06) (*sic*)	37.5	99
Bender et al., Denmark, (32)	≤72 h	123 NICU preterm and term infants with at least 1 clinical sign suggesting EONS: 29 infected, 94 uninfected	(1) Positive blood culture or (2) clinical signs and CRP > 5 mg/dl	(1) Clinical signs and CRP ≤ 5 mg/dl and antibiotic therapy for 3 days or (2) clinical signs, but no antibiotic therapy	Peripheral blood, 0 h (after suspicion of sepsis)	Flow cytometry (LUMINEX)	250 (ROC, specificity ∼95%)	59 (41–75)	94 (87–97)	0.77	76	88
							12 (ROC, sensitivity ∼ specificity)	71	71	0.77	43	89
Resch et al., Austria, (45)	NS	68 NICU preterm and term infants with suspected sepsis: 41 infected, 27 uninfected	(1) Positive blood culture or (2) ≥3 categories of clinical signs, positive sepsis screen and/or risk factors, and antibiotic therapy ≥7 days	Negative blood culture, negative sepsis screen, and antibiotic therapy ≤3 days	Venous or arterial blood, ≤12 h (PNA)	ELISA	≥10 (ROC, NS)	71 (56–82)	67 (48–81)	NA	76	60
							≥60 (ROC, Youden)	54 (39–68)	100 (88–100)	NA	100	59
							≥150 (ROC, NS)	46 (32–61)	100 (88–100)	NA	100	55
Chiesa et al., Italy, (35)	≤48 h	134 NICU preterm and term infants: 19 infected, 115 uninfected	(1) Positive blood culture and clinical signs or (2) ≥3 clinical signs prompting ≥5 days of antibiotic therapy, and historical and clinical risk factors for EONS	Symptomatic infants who had negative body fluid cultures, and were apparently well within 24–48 h and received antibiotic treatment ≤3 days	Cord blood, 0 h (PNA)	ELISA	200 (ROC, Youden)	74 (51–88)	89 (82–93)	NA	NA	NA
					Peripheral blood, 24 h (PNA)		30 (ROC, Youden)	63 (41–81)	71 (62–78)	NA	NA	NA
					Peripheral blood, 48 h (PNA)		20 (ROC, Youden)	53 (32–73)	70 (63–79)	NA	NA	NA
Martin et al., Sweden, (36)	≤48 h	32 NICU preterm and term infants with suspected sepsis: 20 infected, 12 uninfected	(1) Positive blood or CSF culture or (2) abnormal CRP, WBC and ≥1 category of clinical signs (i.e., oliguria, metabolic acidosis, or hypoxemia)	Clinical conditions apparently non-infectious	Peripheral blood, at admission, ≤48 h (PNA)	Chemiluminescence immunoassay	160 (ROC, Youden)	100	70	NA	67	100
Krueger et al., Germany, (34)	≤48 h	136 preterm and term infants: 68 infected, 68 uninfected	(1) Clinical signs and positive blood culture or (2) clinical signs and abnormal laboratory results (CRP, I:T ratio), biological fluids positive for bacteria, or signs of inflammation in placenta	Non-infectious clinical conditions	Cord blood, 0 h (PNA)	Fully automated chemiluminescence immunoassay	80 (ROC, ULC)	87	90	NA	NA	NA
Santana et al., Spain, (14)	NS	31 preterm and term infants: 10 infected, 11 uninfected, 10 healthy controls	≥2 categories of clinical signs, ≥1 abnormal laboratory findings, and positive blood culture	(1) Clinical conditions apparently non-infectious or (2) GA-matched neonates with normal postnatal course through the first month of life	Cord blood, 0 h (PNA)	Chemiluminescence enzymoimmunoassay in the solid phase	100.8 (ROC, NS)	50	87	∼0.5	31	66
Silveira and Procianoy, Brazil, (9)	≤5 days	117NICU infants with suspected sepsis: 66 infected, 51 uninfected	(1) Positive blood and/or CSF culture and ≥3 categories of clinical sepsis or (2) negative cultures and ≥3 categories of clinical sepsis	PROM, but no complete criteria for clinical sepsis, no antibiotic treatment up to discharge from hospital, no hospital readmission (<1 month)	Peripheral blood, 0 h (after suspicion of sepsis), 82.9% at ≤24 h (PNA)	Quantitative sandwich enzyme immunoassay technique (Quantikine)	32 (ROC, NS)	90	43	NA	67.4	78.6
Berner et al., Germany, (46)	≤4 days	136 preterm and term infants, cord blood samples available in 93 infants: 16 infected, 43 uninfected, 35 healthy controls	(1) Positive blood culture or (2) ≥3 categories of clinical signs or laboratory markers	(1) Clinical suspicion but neither positive culture, nor ≥3 categories of clinical signs or	Cord blood, 0 h (PNA)	Double-sandwich enzyme immunoassay (Quantikine)	100 (NA)	87	93	NA	76	97
Døllner et al., Norway, (2)	NS	113 NICU preterm and term infants: 24 infected, 89 uninfected	(1) Positive blood/CSF culture and clinical signs for sepsis/meningitis or (2) negative blood culture, ≥3 categories of clinical signs and abnormal laboratory results (CRP, I:T ratio) or (3) negative blood culture, respiratory symptoms, X-ray consistent with pneumonia, and abnormal laboratory results	Initially suspected of having an infection (not confirmed)	Peripheral blood, at NICU admission or on the next day, >92% <4 days (PNA)	IL-6–dependent mouse hybridoma cell line B13.29 (clone B9), as described by Ng [(15) cite]	20 (NA)	78	71	NA	40	93
							50 (NA)	61	76	NA	38	89
Panero et al., Italy, (33)	≤48 h	60 NICU preterm and term infants: 13 infected, 47 uninfected	Positive blood culture and clinical signs of sepsis	Infants with various types of distress and non-specific abnormal clinical signs who were well within 48–72 h	Venous blood, ≤ 24 h (PNA)	Solid-phase sandwich enzyme-amplified sensitivity immunoassay (Medgenix)	70 (ROC, NS)	69	36	NA	23	81
					Venous blood, ≤24 h (PNA)		200 (ROC, NS)	38	70	NA	26	80
					Venous blood, 24–48 h (PNA)		50 (ROC, NS)	92	96	NA	86	98
Smulian et al., United States, (8)	≤72 h	23 preterm and term infants with suspected EONS: 8 infected, 15 uninfected	(1) Positive blood or CSF culture or (2) clinical signs and ≥laboratory abnormalities (WBC, I:T ratio, PC, ABC, or abnormal spinal tap)	NA	Cord blood (UA), 0 h (PNA)	ELISA (Quantikine)	7 (NA)	88.5	66.6	NA	58.8	91
					Cord blood (UV), 0 h (PNA)	ELISA (Quantikine)	7 (NA)	88.5	93.3	NA	88.5	93.3
Lehrnbecher et al., Germany, (37)	≤48 h	46 NICU preterm and term infants: 13 infected, 33 uninfected	(1) Positive blood culture and ≥3 categories of clinical signs or (2) negative blood culture, ≥3 categories of clinical signs and ≥2 abnormal laboratory results in the first 48 h of life	NA	Cord blood, 0 h (PNA)	Enzyme immunoassay (Dianova-Immunotech)	150 (ROC, NS)	69	91	NA	NA	NA
Messer et al., France, (10)	NS	288 NICU/obstetric unit preterm and term infants: 71 infected (36 infected or probably infected, 35 possibly infected, 217 uninfected	(1) Positive blood and/or CSF culture, clinical signs, and abnormal laboratory results (CRP, WBC) or (2) Negative culture results but ≥3 categories of clinical signs and abnormal laboratory results or (3) negative culture results, <3 categories of clinical signs, abnormal laboratory results that could have another reason, neither exclusion nor confirmation of sepsis possible	Neither clinical nor biological signs of infection	Cord or peripheral blood, NA	ELISA (Hoffmann-La Roche)	100 (ROC, ULC)	83.3	90.3	NA	NA	NA
		Of the 288 infants, 220 were inborn: 39 infected (18 infected or probably infected, 21 possibly infected), 181 uninfected			Cord or peripheral blood, ≤1 h (PNA)			100	92.3	NA	58.8	97
		Of the 288 infants, 254 were sampled within the first 12 h of life: NA			Cord or peripheral blood, ≤12 h (PNA)			100	89	NA	NA	NA

*NA, not available; NS, not specified; UV, umbilical vein; UA, umbilical artery; PNA, postnatal age; NICU, neonatal intensive care unit; CSF, cerebrospinal fluid; CRP, C-reactive protein; WBC, white blood count; PC, platelet count; ABC, absolute band count; EONS, early-onset neonatal sepsis; AUC, area under the curve; PPV, positive predictive value; NPV, negative predictive value; GA, gestational age.*

**TABLE 3:** Characteristics of IL-6 accuracy studies for diagnosing EONS using biomarker combinations.

Author, Year, Country, (Reference)	EONS definition	Recruitment	Reference standard in infected neonates	Reference standard in control neonates	Sample studied, time of sample collection	Test	Biomarker combination	Criterion for positive test	Cutoffs: IL-6 (pg/mL), CRP (mg/L), PCT (ng/mL), TNF-α (pg/mL)	Sensitivity (95% CI), %	Specificity (95% CI), %	AUC	PPV, %	NPV, %
Ebenebe et al., Germany, (4)	≤72 h	1,202 preterm infants with a birth weight < 2,000 g: 67 infected, 115 uninfected	(1) Positive blood culture or (2) CRP ≥ 5 mg/l and ≥ 3 clinical signs	Gestational age and birth-weight matched neonates that did not fulfill criteria of EONS	IL-6: neonatal blood, 0 h (PNA) and maternal blood (CRP), <24 h (before delivery)	IL-6: electrochem iluminescence assay, CRP: particle enhanced immune-nephelometry	IL-6 + CRP	and	IL-6: 40, CRP: 10	49.0	82.4	NA	14.1	96.5
							IL-6 + CRP	Either/or		90.2	43.1	NA	8.6	98.7
					Neonatal blood, 0 h (PNA)	IL-6: electroch emiluminescence assay, CRP: particle enhanced immune-nephelometry	IL-6 + CRP	and	IL-6: 40, CRP: 10	23.4	100	NA	100.0	96.8
							IL-6 + CRP	either/or		75	71.7		13.6	98
Steinberger et al., Austria, (17)	NS	218 NICU preterm infants with risk factors for EONS: 30 infected, 188 uninfected	(1) Positive blood culture or (2) ≥3 categories of clinical signs or (3) ≥1 categories of clinical signs, and ≥2 laboratory abnormalities (CRP, WBC, I:T ratio)	NA	Cord blood	IL-6: ELISA, PCT: LUMItest procalcitonin kit	IL-6 + PCT	and	IL-6: 10, PCT: 0.5	58.8	99.0	0.850 (0.731–0.968)	NA	NA
							IL-6 + PCT	Either/or	IL-6: 15.85, PCT: 0.235 (ROC, Youden)	91.7 (71.2–99.0)	77.1 (67.4–85.0)	0.915 (0.822–1.000)	42.1 (26.3–59.2)	98.7 (92.8–99.8)
Rego et al., Brazil, (42)	NS	144 NICU preterm infants (130 VLBW) presenting RDS during the first 24 h of life: 44 infected, 100 infected	In addition to RDS, (1) ≥ 2 categories of clinical signs, or clinical chorioamnionitis, and positive blood or CSF culture or (2) ≥2 categories of clinical sepsis, or clinical chorioamnionitis, and a hematologic sepsis score >3 or 3) radiographic evidence of pneumonia and a hematologic sepsis score >3	No clinical signs and a hematologic sepsis score <3	Peripheral blood	Chemilum inescence immunoassay system	IL-6 + CRP	and/or	IL-6: 36, CRP: 60 (ROC, maximum sensitivity and specificity >50%)	93 (80–98)	37 (27–48)	NA	41 (31–51)	92 (78–98)
Bender et al., Denmark, (32)	EONS (=72 h)	123 NICU preterm and term infants with at least 1 clinical sign suggesting EONS: 29 infected, 94 uninfected	(1) Positive blood culture or (2) clinical signs and CRP > 5 mg/dl	(1) Clinical signs and CRP ≤ 5 mg/dL and antibiotic therapy for 3 days or (2) clinical signs, but no antibiotic therapy	Blood, 0 h (after suspicion of sepsis)	IL-6: flow cytometry (LUMINEX), PCT: immunol uminometric assay (LUMItest R PCT; BRAHMS Diagnostica, Berlin, Germany)	IL-6 + PCT	Either/or	IL-6: 250, PCT: 25 (specificity of the single marker ∼95%)	71	88	NA	65	91
							IL-6 + PCT	either/or	IL-6: 12, PCT: 5.75 (sensitivity and specificity of the single marker almost identical)	93	46	NA	35	95
Silveira and Procianoy, Brazil, (9)	EONS (≤5 days)	117 NICU preterm and term infants with suspected sepsis: 66 infected, 51 uninfected	(1) Positive blood and/or CSF culture and ≥3 categories of clinical sepsis or (2) negative cultures and ≥3 categories of clinical sepsis	PROM, but no complete criteria for clinical sepsis, no antibiotic treatment up to discharge from hospital, no hospital readmission (<1 month)	Peripheral blood, 0 h (after suspicion of sepsis), 82.9% at ≤24 h PNA	Quantitative sandwich enzyme immunoassay technique (Quantikine)	IL-6 + TNF-α	and/or	IL-6: 32, TNF-α: 12 (ROC, NS)	98.5	NA	NA	60.7	90
Doellner et al., Norway, (2)	NS	113 NICU preterm and term infants: 24 infected, 89 uninfected	(1) Positive blood/CSF culture and clinical signs for sepsis/meningitis or (2) negative blood culture, ≥3 categories of clinical signs and abnormal laboratory results (CRP, I:T ratio) or (3) negative blood culture, respiratory symptoms, X-ray consistent with pneumonia and abnormal laboratory results	Initially suspected of having an infection (not confirmed)	Peripheral blood, on admission to the NICU or on the next day, >92% <4 days (PNA)	IL-6–dependent mouse hybridoma cell line B13.29 (clone B9), as described by Ng [(15) cite]	IL-6 + CRP	and/or	IL-6: 50 pg/ml, CRP: 10 mg/L (NA)	96	74	NA	49	99

*NA, not available; NS, not specified; UV, umbilical vein; UA, umbilical artery; PNA, postnatal age; NICU, neonatal intensive care unit; CSF, cerebrospinal fluid; CRP, C-reactive protein; WBC, white blood count; PC, platelet count; ABC, absolute band count; EONS, early-onset neonatal sepsis; AUC, area under the curve; PPV, positive predictive value; NPV, negative predictive value; GA, gestational age.*

Results of the subgroup analysis are shown in Table 4. Sensitivity was higher in the group of preterm infants (83 vs. 73%), while specificity did not vary among study populations (both 82%). Within the preterm group, a cutoff value ≥30 provided slightly improved sensitivity (84 vs. 80%) and specificity (82 vs. 81%) compared to a lower cutoff value. Within the group of preterm and term infants, a cutoff ≥80 pg/ml led to a drastic increase in specificity (90% vs. 71%) but not sensitivity (both 73%). The sensitivity and specificity in umbilical cord blood was higher than in neonatal peripheral blood, 83 vs. 71% and 85 vs. 77%, respectively. Six (7, 9, 33, 35, 36, 45) of the 12 studies (2, 5, 7, 9, 19, 31, 33, 35, 36, 38, 43, 45) using peripheral blood as sample reported sample collection times earlier than 48 h of life (e.g., ≤6 h, ≤12 h), and studies allowing larger time intervals for sample collection (e.g., <4 days) were grouped under <1 week. Early sample collection (≤48 h) improved the sensitivity of a peripheral blood sample (80 vs. 71% in the overall group), but not the specificity (both 77%). The lowest sensitivity was observed for late sampling (<1 week) of peripheral blood (64%); specificity, however, was not worse than in the early sampling group (77%).

**TABLE 4:** Subgroup analysis of IL-6 diagnostic accuracy studies on EONS.

Subgroup			No. Studies	Pooled sensitivity, %	Pooled specificity, %
Study population	Preterm	All	13	83	82
		<30 pg/ml	6	80	81
		≥30 pg/ml	7	84	82
	Preterm and term	All	18	73	82
		<80 pg/ml	9	73	71
		≥80 pg/ml	9	73	90
Sample and timing	Cord blood	All	18	83	85
		UV	5	87	83
	Peripheral blood	All	12	71	77
		<48 h	6	80	77
		<1 week	6	64	77
Biomarker combinations	IL-6 + CRP		3	84	61

*UV, umbilical vein.*

Six studies reported results from IL-6 combined with either CRP (*n* = 3) or procalcitonin (PCT, *n* = 2) or tumor necrosis factor alpha (TNF-α, *n* = 1) (2, 4, 9, 17, 32, 42). In the three studies analyzing a combination of IL-6 and CRP, cutoff values ranged from 36 to 100 pg/ml and 10 to 60 mg/l, respectively, sensitivities between 75 and 100%, and specificities between 37 and 74% (2, 4, 42). The pooled sensitivity was comparable to that of cord blood IL-6 (3, 6, 8, 14, 17, 20, 28–30, 34, 35, 37, 40, 41, 43, 44, 46) as a single measure (84 vs. 83%), but the pooled specificity was markedly lower (61 vs. 85%).

The assessment of the overall quality of the included studies based on the STARD checklist is summarized in Table 5. All 31 articles included in the meta-analysis were studies of diagnostic accuracy of IL-6, and most resulted from single perinatal centers. Enrollment of patients was based on maternal and prenatal risk factors in seven studies (6, 20, 28, 29, 40, 41, 43) and on clinical signs in further nine studies (4, 7, 17, 19, 31, 32, 34, 38, 44). Three studies included neonates having already been diagnosed with sepsis (4, 38, 44). Almost all of the included IL-6 diagnostic accuracy studies used different reference standards to diagnose EONS and verify index test results leading to differential verification bias. Only three studies used a composite reference standard to exclude sepsis (7, 14, 46). In ten studies, CRP was used as comparator of the index test but also formed part of the reference standard (2, 4, 5, 10, 14, 17, 19, 38, 44, 45). Clinical and demographic data were reported in 22 studies (2–7, 9, 14, 19, 30–36, 38, 40–42, 44, 46). Most studies analyzed birth weight and gestational age as indicators of illness severity, but three studies relied on measures of illness severity that are more objective (7, 35, 42). About a third of the studies (11/31) stated how many neonates failed to undergo the index tests and/or the reference standard (6, 10, 29, 33–35, 38, 40, 42, 44, 45). In the majority of studies, cutoff values were defined *post hoc*. At least 7 studies (6, 8, 10, 19, 35, 41, 46) reported the number, training, and expertise of the persons executing and reading the index test and the reference standard, and 11 studies (2, 6, 8, 10, 19, 29, 31, 35, 42, 43, 46) provided information about masking. Measures of statistical uncertainty (i.e., 95% confidence intervals) and handling of indeterminate results, missing responses, and outliers of index tests were among the least commonly reported items from the STARD checklist (only 8 studies). Fourteen studies provided information regarding methods for calculating IL-6 test reproducibility (3, 7, 9, 10, 14, 20, 30, 34, 35, 41–44).

**TABLE 5:** Quality of IL-6 accuracy studies for diagnosing early-onset neonatal sepsis from 1990 to 2020 according to the STARD criteria (Standards of Reporting Diagnostic Accuracy Studies).

Quality of reporting of IL-6 accuracy studies for diagnosing early-onset neonatal infection		
Category and item no.	YES	NO
**Methods—participants**		
Describe the study population:		
1A. The inclusion and exclusion criteria	22	9
1B. Setting, and locations where data were collected	31	0
Describe participant recruitment:		
2A. Was enrollment of patients based only on clinical signs suggesting infection?	9	22
2B. Were such patients consecutively enrolled?	2	7
2C. Was enrollment of patients based only on maternal risk factors for infection?	7	24
2D. Were such patients consecutively enrolled?	3	4
2E. Were patients identified by searching hospital records?	2	29
2F. Did the study include both patients already diagnosed with sepsis and participants in whom sepsis had been excluded?	3	28
Describe data collection:		
3. Was data collection planned before the index test and reference standard were performed (prospective study)?	15	16
**Test methods**		
Methods pertaining to the reference standard and the index test:		
4A. Was a composite reference standard used to identify all newborns with sepsis, and verify index test results in infected babies?	29	2
4B. Was a reference standard used to exclude sepsis?	14	17
4C. Was a composite reference standard used to identify all newborns without sepsis, and verify index test results in uninfected babies?	3	11
4D. Did the index test or its comparator form part of the reference standard?	10	21
5. Were categories of results of the index test (including cutoffs) and the reference standard defined after obtaining results?	29	2
6. Did the study report the number, training, and expertise of the persons executing and reading the index tests and the reference standard?	7	24
7. Was there blinding to results of the index test and the reference standard?	11	20
**Statistical methods**		
8. Describe the statistical methods used to quantify uncertainty (i.e., 95% confidence intervals)?	5	26
9. Describe methods for calculating test reproducibility	14	17
**Results—participants and test results**		
10A. Describe when the study was done, including beginning and ending dates of recruitment	28	3
10B. Did the study report clinical and demographic (postnatal hours or days, gestational age, birth weight, gender) features in those with and without sepsis?	22	9
10C. Did the study report distribution of illness severity scores in those with and without sepsis?	3	28
11. Report the number of participants satisfying the criteria for inclusion that did or did not undergo the index tests and/or or the reference standard; describe why participants failed to receive either test.	11	20
12. Report a cross-tabulation of the results (including indeterminate and missing results) by the results of the reference standard; for continuous results report the distribution of the test results by the results of the reference standard	23	8
**Results—estimates**		
13. Report measures of statistical uncertainty (i.e., 95% confidence intervals)	5	26
14. Report how indeterminate results, missing responses and outliers of index tests were handled	8	23
15. Report estimates of test reproducibility	14	17

## Discussion

Our meta-analysis including 31 studies with 3,276 infants resulted in a pooled sensitivity of IL-6 of 76% and pooled specificity of 79%. A recent review including 31 studies with 1,448 infants reported a global sensitivity and specificity of 82% (77–86%) and 88% (83–92%), respectively (47). Only 6 studies (7, 9, 32, 36, 40, 45) of this review (47) were also included in our review, partly due to the missing differentiation between early- and late-onset sepsis in their meta-analysis. Differences to our meta-analysis further included selection process, missing differentiation of cord vs. peripheral blood, preterm vs. term infants, influence of pPROM, time of sampling, and combination with other markers. Finally, we included twice as many infants.

We used subgroup analysis to analyze the influence of gestational age in the study population, the cutoff level used, the type of sample, and the time of sample collection. Three studies had to be excluded from the subgroup analysis (32, 33, 42). Reasons were a modification of the cutoff criteria, e.g., to favor a high specificity, so as not to introduce a bias in the subgroup analysis (32, 42), or data provided for illustrative purposes only [Panero et al. (33), data for postnatal day 1]. Some groups provided data for different scenarios, e.g., analyzing different samples or varying the cutoff level. For the subgroup analysis of preterm vs. preterm and term infants, each study was only included once, so as not to introduce the same study population multiple times within the same subgroup analysis. This was done by choosing the scenario yielding the best results, or, if subgroups were analyzed, the one including the whole study population. One study did not specify whether they used cord or peripheral blood and was excluded from the subgroup analysis regarding the type of sample (4). Subgroup analysis showed that sensitivity of IL-6 was higher in the group of preterm infants compared to the mixed group of preterm and term infants (83 vs. 73%), while specificity did not vary among study populations (both 82%). Even though a wide cutoff range of 11–300 pg/ml resulted from the included publications, we found that a cutoff value ≥30 provided only slightly improved sensitivity (84 vs. 80%) and specificity (82 vs. 81%) compared to a lower cutoff value in a group of preterm infants. These findings are in agreement with Qiu et al. (25). In the group of preterm and term infants, however, a cutoff of ≥80 pg/ml led to a drastic increase in specificity (90 vs. 71%) but not sensitivity (both 73%). In general, sensitivity and specificity values were found to vary greatly among different studies even for the same cutoff value, thus suggesting a different source of heterogeneity. We found a higher pooled sensitivity (83 vs. 71%) and specificity (85 vs. 77%) for umbilical cord blood compared to peripheral blood samples. In contrast, Qiu et al. (25) found a higher sensitivity and specificity of IL-6 in peripheral blood within a population of pPROM infants. Our results revealed improved sensitivity (80 vs. 71%) and constant specificity (77%) of early sampling within the first 48 h from peripheral blood.

EONS was more frequently observed in infants with pPROM than in premature infants with intact membranes (38 vs. 10%, *p* = 0.001) (29). Infants with pPROM had increased cord blood IL-6 levels, which were significantly higher in neonates who developed EONS and thus had a higher predictive value than clinical signs of chorioamnionitis (29). Another group found that cord blood IL-6 but not funisitis in women complicated with pPROM was an independent predictor for the occurrence of EONS (6). A meta-analysis investigating IL-6 as a diagnostic tool after pPROM included nine studies and reported a pooled sensitivity of 85% and specificity of 88% (25). The cutoff values ranged between 7.8 and 108.5 pg/ml correlating with sensitivities between 46 and 95% and specificities between 63.3 and 100%. Two-thirds of studies reported cord blood IL-6 values and mixed populations of preterm and term infants.

Six studies included analyzed biomarker combinations (2, 4, 9, 17, 32, 42). Messer et al. (10) stated that IL-6 appeared to be an ideal marker before the age of 12 h and in combination with CRP, thereafter leading to a sensitivity of 100%. However, this was hardly surprising since an elevated CRP level was one of the classification criteria. The combination of IL-6 > 36 pg/ml (0 h) and/or CRP > 60 mg/l (24 h) was able to increase sensitivity (93 vs. 82%). However, the specificity remained low (37%). Another study combined IL-6 > 250 pg/ml and PCT > 25 ng/ml resulting in a sensitivity of 71% and a specificity of 88% at the time of sepsis suspicion (32). Steinberger et al. (17) using cord blood cutoff values of IL-6 > 15.85 pg/ml and PCT > 0.235 ng/ml reported sensitivity and specificity of 91.7 and 77.1%, respectively, with an excellent AUC of 0.915. Silveira et al. (9) found that IL-6 > 32 pg/ml and TNF-α > 12 pg/ml had a sensitivity of 98.5%. Unfortunately, they did not report the specificity of their biomarker combination. Some studies suggested the use of three inflammatory markers (17, 31, 38). Using single-parameter analysis, Steinberger et al. (17) suggested a combined use of cord blood PCT and IL-6 with serial determinations of CRP over the first days of life to rule out infection (17). Other combinations like hs-CRP, PCT, and IL-6 (38) or presepsin, PCT, and one proinflammatory cytokine, either IL-6 or IL-8 (31), were found to be superior to the individual markers. Using an early and sensitive marker like IL-6 for screening, and confirming sepsis suspicion with a late and specific marker like CRP, measured a few hours later, is effective in diagnosing EONS (7). The potential of such combinations, however, might rather lie in their simultaneous measurement at sepsis suspicion (32). Their counteractive dynamics suggest using an either/or combination (4). Findings suggest that it is possible to define high cutoff values, increasing the specificity of the single markers, because a satisfactory sensitivity can be reached over the biomarker combination (2, 32, 42).

Chiesa et al. (22) described the quality of IL-6 diagnostic accuracy studies as suboptimal, with missing information on key elements like design, conduct, analysis, and interpretation of test accuracy. Study designs like non-consecutive sampling of patients, retrospective data collection, and identification of patients by searching hospital records are prone to spectrum bias (22). Reporting the actual dates of when the study was performed allows the reader to consider any technological advances that have taken place in the meantime. If more than one reference standard is used to verify results of the index test, incorrectly treating their results as equivalent will lead to differential verification bias (48). Reported estimates of diagnostic accuracy are on average 60% higher than those found in studies that used a single reference standard (49). However, reference standards are not interchangeable as they may not have the same degree of error and may not identify the same segment of the disease (22). Consequently, the decision to not use positive blood culture as sole standard for diagnosing EONS in diagnostic accuracy studies has been described as arguable (4) and small numbers of culture proven sepsis cases within the study population are usually reported as limitation of the study (5, 19, 20, 32). The use of a mixed study population of culture-positive and clinical sepsis cases, however, is supported by the fact that the positive culture rate is extremely low in patients with EONS (19). This holds especially true for infants born to mothers who received antenatal or intrapartum antibiotics (43). Many studies comparing the two diagnostic subgroups found that IL-6 levels did not differ significantly (2, 10, 35, 46, 50, 51). FIRS might be associated with neonatal sepsis (28) but also with a neonatal systemic inflammatory response, which manifests as clinically suspected neonatal sepsis with negative blood and cerebrospinal fluid cultures (52). Adding a group of sick neonates without infection, in which IL-6 was also significantly increased, to healthy controls lessened the diagnostic value of IL-6 (14).

Some studies reported data of a group of patients in whom the applied diagnostic criteria resulted non-conclusive, so that sepsis could neither be excluded nor confirmed (2, 10, 33–35). IL-6 levels in these neonates were found to be higher than in uninfected sick controls, but lower than in neonates with infection (2). Termed uncertain sepsis (33, 35), infection unlikely (34), or mixed group (2), those groups were mostly excluded from study analysis and the determination of cutoff values. While not all studies provided this information (34), it was generally assumed in this analysis.

In diagnostic accuracy studies, it is of utmost importance to describe the populations from which patients and patient controls originated as well as the severity of sepsis within the patient group (22). Illness severity may alter the diagnostic value of IL-6 (35), and the use of illness severity scores has been included into the STARD checklist for assessing the quality of IL-6 accuracy studies (22). Chiesa et al. (35) used the Score for Neonatal Acute Physiology (SNAP) and its Perinatal Extension (SNAP-PE) (53). High IL-6 values in infants with EONS were independent of illness severity in contrast to uninfected infants in which higher IL-6 levels correlated with higher SNAP scores. Similar results were reported by Labenne et al. (7). Messer et al. (10) found no correlation between the magnitude of IL-6 levels and the severity of infection. These findings indicate that illness severity does not influence IL-6 levels in infected infants but leads to increased levels in uninfected infants, affecting the specificity of a diagnostic test relying on IL-6.

If the result of the index test influences the decision to order the reference test, measures of diagnostic accuracy will be biased (54). Incorporation bias occurs if the index test or the comparator of the index test form part of the reference standard (54, 55). This gives the person interpreting the results of the index test or its comparator some knowledge of the results of the reference standard (22). CRP was part of the reference standard for sepsis diagnosis in most of the included studies. Not only does this fact distort the diagnostic abilities of CRP when used as a comparator of the index test, but also biomarker combinations including CRP and markers related to CRP are biased (32). Therefore, blinding to both the index test and the knowledge of its outcome should be performed to avoid test review and diagnostic review bias (22).

In the included studies, cutoffs were mostly defined *post hoc* using ROC analyses and Youden’s index. While Youden’s index has the advantage of being a single measure, it loses the distinction between the sensitivity and specificity of a test. So do other error-based measures like the area under the ROC curve, an estimator of the overall accuracy of a test (17). Defining the cutoffs after the results are obtained reduces the likelihood that another study will replicate the findings (22).

### Strengths of the Study

The type of sepsis has been identified as a significant source of heterogeneity (*p* = 0.0351) through a subgroup analysis conducted by Qiu et al. (25). This was apparent even though their subgroups were formed by a group of early-onset sepsis cases and a mixed (i.e., early/late-onset) sepsis group. We eliminated this factor by including only cases of EONS in our meta-analysis.

### Limitations of the Study

There are several important limitations to our systematic review and meta-analysis. First, we limited our database search to PubMed, which might have yielded a biased sample of primary studies and, thus, may influence the accuracy of summary effects. We did, however, check the reference lists of included studies and other important systematic reviews, to include other relevant studies. We did not include unpublished data and data reported in abstract form, which may result in publication bias. To investigate possible sources of heterogeneity within IL-6 diagnostic accuracy studies, we included a heterogeneous group of studies in this meta-analysis. While this gave us a sufficient number of studies (≥5 studies in each subgroup, with the exception of biomarker combinations) for meaningful subgroup analysis of multiple possible influencing factors, it might have compromised the precision of our study, due to remaining sources of heterogeneity within the subgroups. Exploration of a specific source of heterogeneity within an otherwise homogenous subgroup might be subject for future research. The small number of studies looking at biomarker combinations limited our attempt to give more information on their potential, as it did not allow for subgroup analysis. Finally, initial data selection and collection were performed by reviewer JE only; the final decision however was based on a discussion with reviewer BR and resolved by consensus.

## Conclusion

We identified 31 studies on IL-6 diagnostic accuracy for EONS diagnosis between 1990 and 2020 including 3,276 infants. The range of IL-6 sensitivities and specificities in neonatal samples was 42.1–100% and 43–100% with median values of 83 and 83.3%, respectively. IL-6 accuracy was better in preterm infants than in mixed term and preterm infants. The sensitivity and specificity in umbilical cord blood were higher than in neonatal peripheral blood, 83 vs. 71% and 85 vs. 77%, respectively. Diagnostic accuracy in peripheral blood was higher if blood was drawn within the first 48 h. The combination of IL-6 and CRP had a sensitivity in the range of cord blood IL-6 as single measure (84 vs. 83%), but far lower specificity (61 vs. 85%). Finally, quality assessment by the STARD criteria revealed poor quality of the majority of studies; thus, we need better designed, prospective, multicenter investigations on IL-6 and its use for the prediction of EONS.

## Data Availability Statement

The original contributions presented in the study are included in the article/supplementary material, further inquiries can be directed to the corresponding author/s.

## Author Contributions

JE and BR contributed to conception and design of the study and wrote sections of the manuscript. JE performed the database search, study selection, data extraction, and statistical analysis and wrote the first draft of the manuscript. Both authors contributed to manuscript revision, read, and approved the submitted version.

## Conflict of Interest

The authors declare that the research was conducted in the absence of any commercial or financial relationships that could be construed as a potential conflict of interest.

## Publisher’s Note

All claims expressed in this article are solely those of the authors and do not necessarily represent those of their affiliated organizations, or those of the publisher, the editors and the reviewers. Any product that may be evaluated in this article, or claim that may be made by its manufacturer, is not guaranteed or endorsed by the publisher.
